# Role of Chitinase-3-like Protein 1 in Cardioprotection and Angiogenesis by Post-Infarction Exercise Training

**DOI:** 10.3390/biomedicines10051028

**Published:** 2022-04-29

**Authors:** Zhuo Li, Fangnan Wu, Lei Xi, Zhenjun Tian

**Affiliations:** 1 Institute of Sports and Exercise Biology, School of Physical Education, Shaanxi Normal University, Xi’an 710119, China; lzhuoyx@163.com; 2 School of Life Science and Technology, Xi’an Jiaotong University, Xi’an 710049, China; wfn9699@stu.xjtu.edu.cn; 3 Pauley Heart Center, Department of Internal Medicine, Virginia Commonwealth University, Richmond, VA 23298, USA

**Keywords:** angiogenesis, cardioprotection, cardiac remodeling, exercise, cytokine, myocardial infarction

## Abstract

Chitinase-3-like protein 1 (CHI3L1) is a myokine involving tissue remodeling and inflammatory processes. CHI3L1 and its receptor protease-activated receptor 2 (PAR2) are induced by exercise in skeletal muscles. However, it remains unknown if CHI3L1/PAR2 signaling also mediates exercise-induced cardioprotection after myocardial infarction. Twenty-four adult male rats were divided into three groups (n = 8/group), receiving: (1) a sham operation; (2) permanent ligation of left anterior descending coronary artery; and (3) post-MI exercise training with one-week adaptive treadmill exercise for seven days followed by four weeks of aerobic exercise. Left ventricular systolic and end-diastolic pressure indices were measured and cardiac fibrosis, and angiogenesis were examined. Furthermore, HUVEC cells were treated in vitro with AMPK agonist—AICAR (a putative pharmacological memetic of exercise), recombinant human CHI3L1, PAR2 receptor blocker (AZ3451), and PI3K inhibitor (LY294002), respectively. We found that post-MI exercise significantly upregulated CHI3L1, PAR2, pPI3K/PI3K, pAKT/AKT, pERK/ERK, improved cardiac function, and diminished fibrosis. AICAR increased HUVEC tubules formation and upregulated CHI3L1 and PAR2 and these changes were attenuated by PAR2 blocker. In conclusion, post-MI exercise training can effectively activate CHI3L1/PAR2 signaling, which led to the improved myocardial function and enhanced cardiac angiogenesis in the infarcted heart.

## 1. Introduction

Ischemic heart attack, i.e., myocardial infarction (MI), is caused by reduced or completely interrupted blood flow to myocardium and the resultant severe hypoxia/ischemia in cardiac tissues often lead to necrosis and apoptosis in cardiac cells including cardiomyocytes [1,2], myocardial collagen hyperplasia [3], and eventually cardiac dysfunction. The search for effective strategies of reduction and rehabilitation of detrimental outcomes of MI has been a focal area of research in cardiovascular science and medicine. In particular, studies have confirmed that appropriate exercise training in post-MI recovery period can improve cardiac function [4], decrease the degree of myocardial fibrosis [5], and, in turn, reduce the recurrence rate and mortality of the patients suffering with MI [6]. Post-MI exercise training also inhibited apoptosis, promoted angiogenesis, and enhanced cardiac function in rats [7]. Nevertheless, the precise molecular targets and cell signaling mechanisms of post-MI exercise-induced cardioprotection remain elusive. Previous studies from our laboratory have demonstrated the role of FSTL1 [8] and SIRT1/PGC-1α/PI3K/Akt signaling [9] in mediating the beneficial effects afforded by post-MI exercise.

Chitinase-3-like protein 1 (CHI3L1, also known as YKL-40 or cartilage glycoprotein 39), is a secreted glycoprotein with chitin-binding activity with weak chitinase activity [10]. CHI3L1 may be secreted by adipocytes, epithelial cells, chondrocytes, tumor cells, as well as macrophages, and is present in various vital organs such as liver, kidney, heart, and skeletal muscle [11,12,13]. CHI3L1 can bind to its receptor PAR2 (protease-activated receptor 2) or IL-13Rα2 to promote mitosis and cell growth, while antagonize apoptosis [14]. Furthermore, CHI3L1 plays an important role in regulating acute and chronic inflammation, tumorigeneses, extracellular matrix remodeling, etc. [15,16,17,18]. Notably, the circulating level of CHI3L1 (i.e., YKL-40) has been investigated as a potential predictive biomarker for prognosis of cancers [19] as well as acute coronary syndrome [20] and MI [21,22,23]. YKL-40 levels have been found to be associated with all-cause and cardiovascular mortality and are elevated in patients with type 1 or type 2 diabetes [24]. In addition, muscular exercise has been shown to regulate the expression of CHI3L1 and PAR2 in skeletal muscles and stimulate skeletal muscle cell proliferation and hypertrophy via activation of p38 MAPK and Akt signaling pathways [25]. However, whether or not CHI3L1 is involved in cardioprotection of exercise against MI-induced myocardial injury remains unknown. In this context, the present study was designed to investigate the possible involvement of CHI3L1/PAR2 signaling in cardioprotection induced by post-MI exercise training.

## 2. Materials and Methods

### 2.1. Animals and Experimental Groups and Protocols

Three-month-old male Sprague Dawley rats were purchased from the Animal Center of Xi’an Jiaotong University, China. The animal experiment protocol was approved by the Institutional Animal Care and Use Committee of Shaanxi Normal University (Xi’an, China).

**Figure 1:**
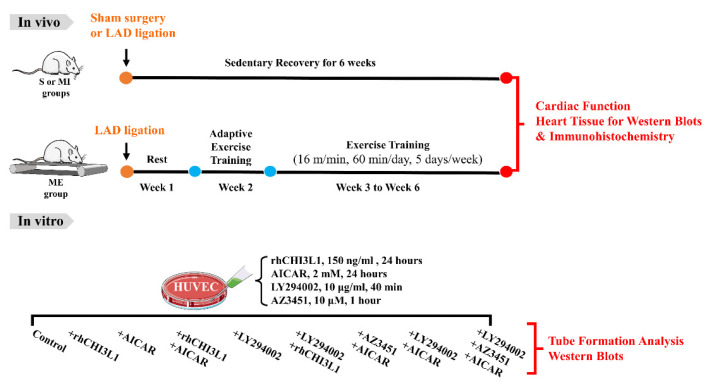
Schematic description of the study protocols in adult male rats involving induction of myocardial infarction (MI) through permanent ligation of left anterior descending coronary artery and post-MI exercise training that were carried out one week after the MI surgery and lasted five weeks, i.e., one-week adaptive exercise followed by aerobic exercise training sessions for additional four weeks. A parallel series of in vitro study in human umbilical vein endothelial cells (HUVEC) was also performed under the illustrated treatment conditions.

As illustrated in Figure 1, 24 rats were randomly divided into three groups (*n* = 8/group), received (1) sham surgery (Group S); (2) myocardial infarction induced by permanently ligation of left anterior descending coronary artery (LAD) (Group MI); and (3) post-MI aerobic exercise training (Group ME). All animals were fasted overnight before the surgical procedures and were anesthetized with intraperitoneal (i.p.) injection of sodium pentobarbital (30 mg/kg). The rats in the post-MI exercise group (ME) underwent one week of post-MI adaptive exercise training (30 min per day for 5 days; treadmill speed 10–15 m/min) on a motorized rodent treadmill (Model ZH-PT, Anhui Zhenghua Technology Co., Huaibei, China) and subsequently 4 weeks of exercise training (60 min per day, 5 days per week for 4 weeks; treadmill speed 16 m/min).

### 2.2. In Vivo Cardiac Function Measurement

The rats were fasted overnight and anesthetized with pentobarbital (30 mg/kg) and a pressure catheter (polyethylene tubing #SP0109, AD Instruments, Sydney, Australia) was inserted into the right carotid artery and advanced into the left ventricular cavity. The pressure parameters were recorded in each of the anesthetized rats via a pressure transducer for 5 min and analyzed a randomly selected 1 min segment of the recorded data to determine various hemodynamic indexes, such as left ventricular systolic pressure (LVSP), left ventricular end-diastolic pressure (LVEDP), and positive maximum or negative minimum rate of pressure change in the left ventricle (+dP/dt or −dP/dt).

### 2.3. Heart Sample Collection and Processing for Histology Staining

After the hemodynamic analysis, the hearts were quickly removed via thoracotomy, washed with cold saline, weighed, and frozen in liquid nitrogen (*n* = 5/group) and then transferred to a −80 °C freezer for storage until the subsequent molecular biology analysis. Additional 3 hearts per group were fixed in 10% neutral formaldehyde solution for morphological examination. The heart tissue samples were processed with conventional paraffin embedding and cut into sections of 5 μm thickness for Masson staining and further examination of histological characters under optical microscopy, such as myocardial collagen volume percentage (CVF%).

### 2.4. In Vitro Study in HUVEC Cells

Human umbilical vein endothelial cells (HUVEC) were purchased from American Type Culture Collection (ATCC) and cultured in DMEM with 10% FBS at 37 °C in a CO_2_ incubator (Thermo Forma 311, Thermo Scientific, Waltham, MA, United States). The following compounds were administered to the cells for incubation in various time duration: i.e., (1) AMPK agonist (AICAR, 2 mM for 24 h), which was used to mimic the metabolic effects of physical exercise [26]; (2) PAR2 receptor blocker (AZ3451, 10 μM for 1 h); (3) PI3K inhibitor (LY294002, 10 μg/mL for 40 min), and (4) rhCHI3L1 (150 ng/mL for 24 h).

### 2.5. HUVEC Tube Formation Analysis

For the tubule formation assay, the 24-well plate, the pipette tip, and Matrigel (Cat# 354248, BD Bioscience, Billerica, MA, USA) were pre-cooled or dissolved at 4 °C. A precooled tip was used to add approximately 300 µL of Matrigel into a 24-well plate in cold room in order to prevent gelation. The 24-well plate was placed in an incubator for 30 to 60 min. 500 µL of the cell suspension (2 × 10^5^ cells/mL) was added to each well in the plate, which was placed in an incubator for static culture.

### 2.6. Protein Extraction and Western Blotting

50 mg of heart tissues removed from the border zones of infarct area were cut and homogenized in RIPA buffer (Cat# P0013B, Beyotime Biotechnology, Shanghai, China) added with protease inhibitor cocktail (Cat# 78430, Thermo Scientific, Waltham, MA, USA), and centrifuged at 12,000 rpm for 30 min at 4 °C. The supernatant was collected and the protein concentration was quantified using a BCA protein assay (Cat# P0012, Beyotime Biotechnology, Shanghai, China). For western blotting experiments, protein samples were first separated with SurePAGE gels (Cat# M00656, GenScript, Nanjing, China) and transferred to a nitrocellulose membrane (Millipore, Burlington, MA, USA), which was incubated with one of the following primary antibodies in the indicated concentrations: CHI3L1 (1:500, #ab77528, Abcam, Cambridge, MA, USA), PAR2 (1:500; #YN2681, Immunoway Biotechnology, Plano, TX, USA), PI3K (1:1000, #4249, Cell Signaling Technology, Danvers, MA, USA), p-PI3K (1:500; #17366, Cell Signaling Technology, Danvers, MA, USA), ERK (1:1000, #4695, Cell Signaling Technology, Danvers, MA, USA), pERK (1:500, #4370, Cell Signaling Technology, Danvers, MA, USA), eNOS (1:800; #ab16798, Abcam, Cambridge, MA, USA), and GAPDH (1:3000, #TDY042, TDY biotech, Beijing, China).

### 2.7. RNA Extraction and Quantitative Reverse Transcription PCR (RT-qPCR)

Total RNA were extracted from rat cardiac tissues using Trizol reagent (B511311, Sangon Biotech, Shanghai, China). cDNA was obtained using the PrimeScript™ RT reagent Kit (#RR037A, TaKaRa, Kusatsu, Japan). CHI3L1 was tested using miScript SYBR Green PCR Kit (#DRR041B, TaKaRa, Kusatsu, Japan). RT-qPCR reactions were performed with a quantitative PCR instrument (Bio-Rad model CFX96, Hercules, CA, USA). Forward and reverse primers of CHI3L1 and GAPDH were provided by Sangon Biotech (Shanghai, China). Quantification of relative gene expression was calculated by the comparative Ct method (2-ΔΔct) as described by the manufacturer.

The primer sequences used are the follows:

*Chi3l1*  Forward: 5′-TACGGCATGCTCAACACACT-3′
  Reverse: 5′-TGCCCATCACCAGCTTACTG-3′
*gapdh*  Forward: 5′-TGACTTCAACAGCGACACCCA-3′
  Reverse: 5′-CACCCTGTTGCTGTAGCCAAA-3′

### 2.8. Immunofluorescence Analysis and Image Processing

Paraffin-embedded heart sections were dewaxed, washed with PBS, processed for antigen retrieval, washed with PBS, blocked with 5% BSA (37 °C, 1 h), and then incubated at 4 °C overnight. The next day, the sections were washed three times with PBS under dark condition and incubated with secondary antibody at 37 °C for 1 h, and then washed three times with PBS, prior to the fluorescence microscopy.

A gel imaging system (Tanon-5200Multi, Los Altos, CA, USA) were used to obtain the images of the protein bands following western blotting and the optical density was quantified using ImageJ software (version 1.50i, Wayne Rasband, National Institutes of Health, Bethesda, MD, USA). The Masson’s trichrome-stained and immunofluorescence images were processed using Image-Pro Plus (version IPP 6.0, Media Cybernetics, Rockville, MD, USA).

### 2.9. Blood Sample Collection and Measurement of Serum Levels of CHI31L with ELISA

Blood samples were collected from abdominal aorta of the rats and placed at 37 ℃ for 60 min prior to centrifugation at 3000 rpm for 10 min to collect serum fraction. The expression levels of CHI31L in the serum samples were detected using a CHI3L1 ELISA Kit manufactured by Abcam (Item# ab238262, Cambridge, MA, USA). The optical density (OD) value of each sample well was measured with a BioTek Epoch microplate analyzer (Winooski, VT, USA) at the wavelength of 450 nm and was used for calculating the individual expression levels of CHI3L1 in serum.

### 2.10. Statistical Analysis

The data were analyzed by one-way ANOVA followed by Tukey’s multiple comparisons test using GraphPad Prism 8 (version 8.0.2, San Diego, CA, USA). The data are presented as Mean ± Standard Deviation (SD). *p* value of < 0.05 is considered statistically significant.

## 3. Results

### 3.1. Post-MI Exercise Reduces Myocardial Fibrosis and Improves Cardiac Dysfunction

**Figure 2:**
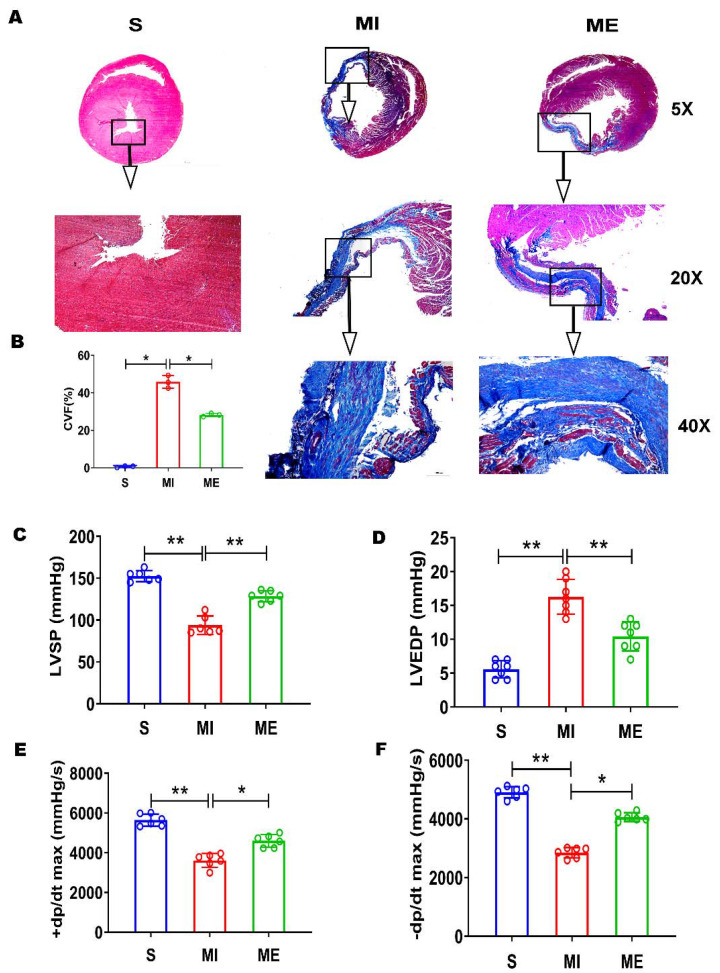
Post-myocardial infarction (MI) aerobic exercise training alleviates myocardial pro-fibrotic remodeling and improves left ventricular (LV) function in the rat heart. (**A**) Representative pictures of Masson staining of paraffin sections in the rat heart (in 5×, 20×, 40× microscopic magnification, respectively), which show that normal myocardial fibers are stained in red, whereas collagen fibers are stained in blue color. (**B**) Bar graph shows quantified myocardial collagen volume (CVF%, *n* = 3/group), which equals Blue area/(Blue area + Red area) × 100. (**C**–**F**) Bar graphs show averaged systolic (LVSP, +dp/dtmax) and diastolic (LVEDP, −dp/dtmax) LV function parameters (*n* = 6/group). *Abbreviations:* S, sham control group; MI, myocardial infarction group; ME, post-MI exercise trained group. Symbol * indicates *p* < 0.05 and ** indicates *p* < 0.01 between the relevant groups that are compared.

Masson staining showed that myocardial fibrosis occurred more in MI group than S group, to form scar tissue. Post-MI exercise training lowered myocardial collagen volume (CVF%) significantly (*p* < 0.01, ME versus MI group; Figure 2A,B). Hemodynamic measurements showed that LVEDP was significantly elevated in MI group (*p* < 0.05, versus S group; Figure 2D), whereas ±dP/dt max and LVSP significantly decreased (*p* < 0.05, Figure 2C,E,F). The MI-elevated LVEDP was significantly reduced by exercise in ME group (*p* < 0.05, Figure 2D), whereas the MI-caused depression of ±dP/dt max and LVSP was alleviated by post-MI exercise training (*p* < 0.05 ME versus MI group; Figure 2C,E,F).

### 3.2. Post-MI Exercise Promotes Myocardial Angiogenesis

**Figure 3:**
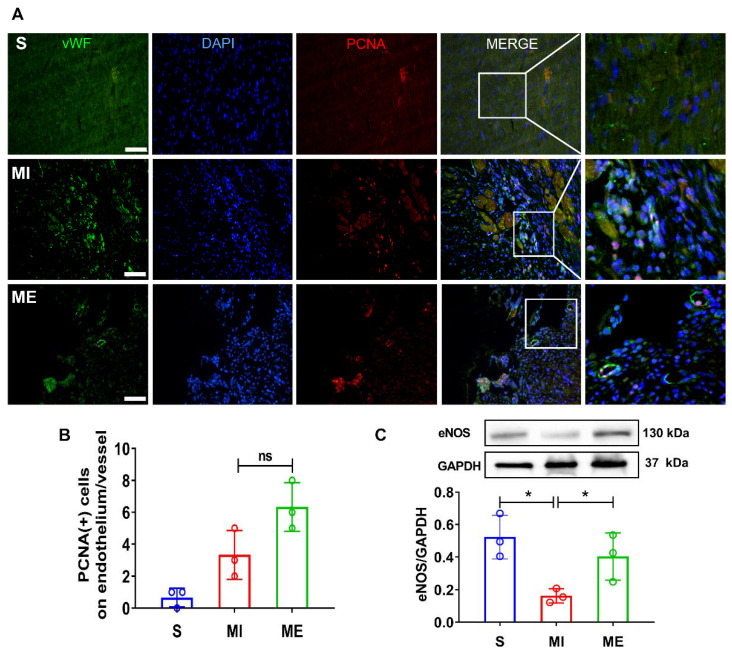
Post-myocardial infarction (MI) aerobic exercise training promotes cardiac angiogenesis and upregulates endothelial nitric oxide synthase (eNOS) in adult male rats. (**A**) Representative pictures of immunofluorescent staining of proliferating cell nuclear antigen (PCNA)/von Willebrand factor (vWF) (markers of angiogenesis) in rat heart paraffin sections. Whereas vWF indicates green fluorescence-labeled vascular endothelium, DAPI is blue fluorescence-labeled nuclei and PCNA is red fluorescence-labeled neonatal nuclei (scale bar = 200 µm; *n* = 3/group). (**B**) Bar graph shows quantitative results of the respective intensity of immunofluorescence. (**C**) Representative pictures of western blots for detecting cardiac expression levels of the angiogenesis-related protein—eNOS and bar graph of the averaged densitometry results of eNOS protein in rat myocardium (*n* = 3/group). *Abbreviations*: S, sham control group; MI, myocardial infarction group; ME, post-MI exercise trained group. Symbol * indicates *p* < 0.05 between the relevant groups that are compared.

Since myocardial parenchymal components are impaired after MI and blood supply to myocardium is blocked or restricted, post-MI exercise may counter these pathological changes via promoting angiogenesis in the heart. Our present study used von Willebrand factor (vWF) to label vascular endothelial cells and proliferating cell nuclear antigen (PCNA) to label proliferating nuclei (Figure 3A). The numbers of PCNA and vWF double-positive cells increased in MI and ME groups as compared with S group, but the difference did not reach statistical significance (Figure 3B), indicating a trend for enhanced myocardial angiogenesis. On the other hand, cardiac expression of eNOS protein was significantly downregulated in MI group as compared with S group and restored in ME group (*p* < 0.05, Figure 3C).

### 3.3. Post-MI Exercise Activates CHI3L1/PAR2-PI3K-AKT/ERK Signaling Pathways in Heart

**Figure 4:**
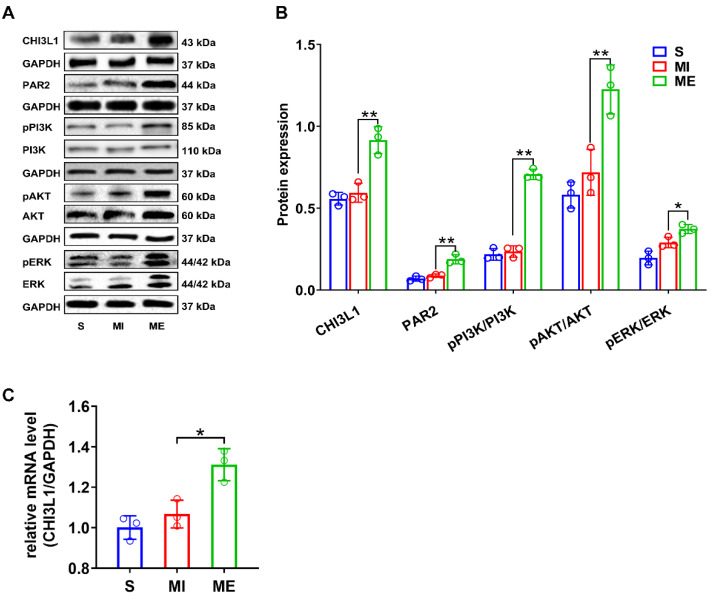
Post-myocardial infarction (MI) aerobic exercise training activates CHI3L1/PAR2-PI3K-AKT/ERK signaling pathway in the rat heart. (**A**) Representative pictures of western blotting indicating the protein levels of the relevant signaling molecules. (**B**) Quantitative analysis for CHI3L1 and PAR2 protein levels, and phosphorylation levels of PI3K, Akt and ERK in heart tissues (*n* = 3/group). (**C**) Relative mRNA level of CHI3L1 in myocardium assessed by RT-qPCR (*n* = 3/group). *Abbreviations*: S, sham control group; MI, myocardial infarction group; ME, post-MI exercise trained group. Symbol * indicates *p* < 0.05 and ** indicates *p* < 0.01 between the relevant groups that are compared.

Whereas MI perse did not cause significant change in cardiac expression of CHI3L1 and its receptor PAR2 versus controls in S group, post-MI exercise training significantly upregulated CHI3L1 and PAR2 protein expression in the heart (*p* < 0.01, ME versus MI group; Figure 4). Furthermore, the similar significant increase in the ratios of pPI3K/PI3K, pAKT/AKT and pERK/ERK was also found in ME group MI group (*p* < 0.05 versus MI group; Figure 4), suggesting activation of the PI3K-AKT/ERK signaling pathways by post-MI exercise.

### 3.4. Post-MI Exercise Training Significantly Upregulated Serum Levels of CHI3L1

**Figure 5:**
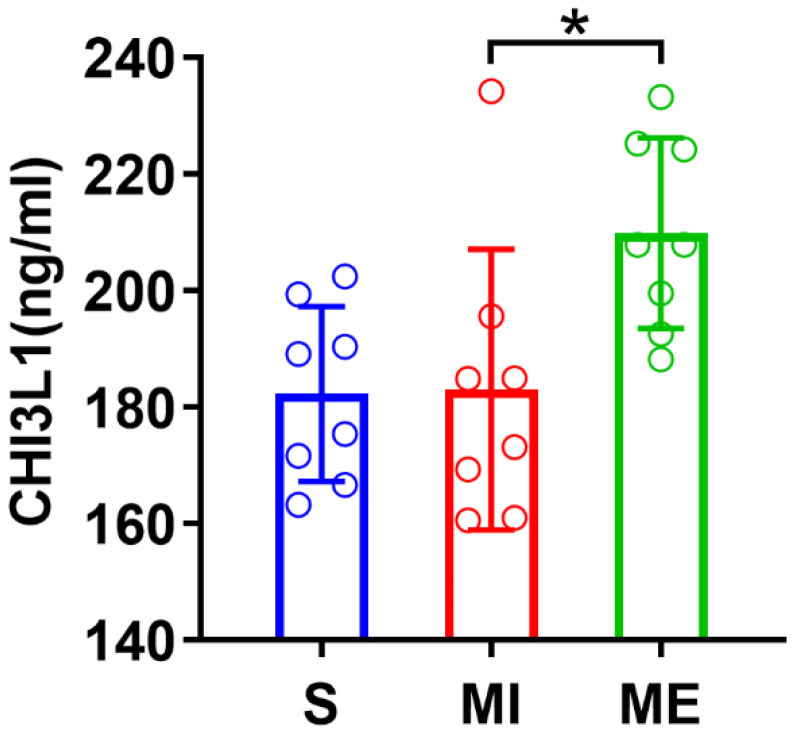
Quantified levels of CHI3L1 from rat serum samples (*n* = 8/group) assessed by ELISA. *Abbreviations*: S, sham control group; MI, myocardial infarction group; ME, post-MI exercise trained group. Symbol * indicates *p* < 0.05 when comparing groups MI and ME.

The ELISA-detected expression levels of CHI3L1 in serum samples (*n* = 6/group) from the MI group was not significantly different from the S group (Figure 5), whereas the CHI3L1 levels were significantly upregulated in ME as compared with MI group (*p* < 0.05). These results indicated that post-MI exercise training led to an enhanced circulating levels of CHI3L1 and most likely originated from the exercising skeletal muscles.

### 3.5. CHI3L1 or AICAR Promotes HUVEC Tubule Formation

**Figure 6:**
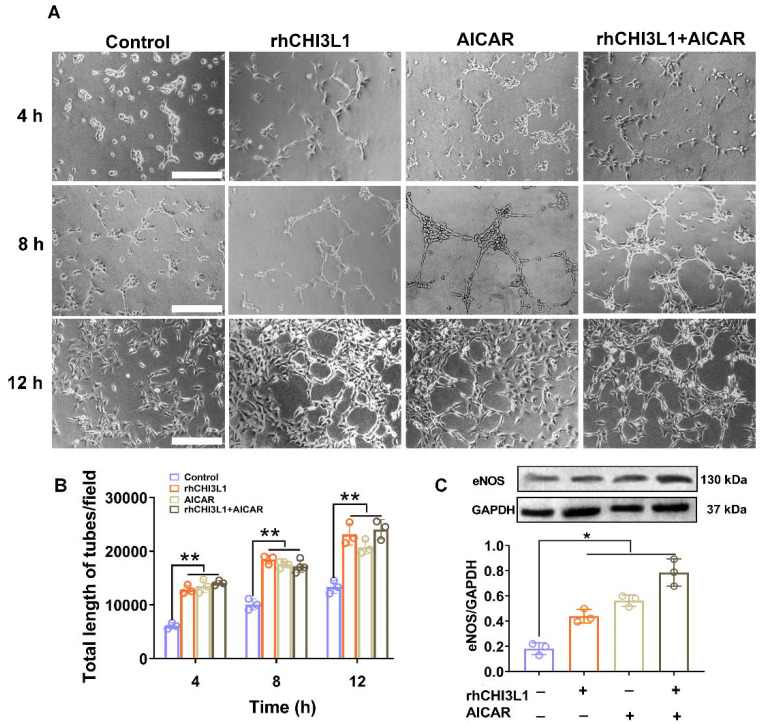
Pro-angiogenesis effects of rhCHI3L1 and AICAR in human umbilical vein endothelial cells (HUVEC). (**A**) Representative microscopic images demonstrating HUVEC tubule formation (scale bar = 500 µm). (**B**) Bar graph shows quantitative results of HUVEC tubule formation (*n* = 3/group). (**C**) Representative pictures of western blotting for detection of expression levels of eNOS, an important pro-angiogenic protein in HUVEC cells under various treatments along with the bar graph showing densitometric results of the eNOS protein band normalized with GAPDH, a housekeeping control protein (*n* = 3/group). *Abbreviations*: rhCHI3L1, recombined human chitinase-3-like protein 1; AICAR, 5-aminoimidazole-4-carboxamide-1-β-D-ribofuranoside; eNOS, endothelial nitric oxide synthase. Symbol * indicates *p* < 0.05 and ** indicates *p* < 0.01 between the relevant groups that are compared.

In cultured HUVECs under logarithmic growth phase, incubation with rhCHI3L1 or AICAR (2 mM) significantly promoted HUVEC tubule formation at various time points (4, 8, and 12 h) as compared with normal control group (*p* < 0.01, Figure 6A,B). rhCHI3L1 or AICAR also significantly increased eNOS protein expression as compared with control group (*p* < 0.05, Figure 6C).

### 3.6. CHI3L1 or AICAR Promotes Angiogenesis through the PI3K-AKT/ERK Pathway and PAR2

**Figure 7:**
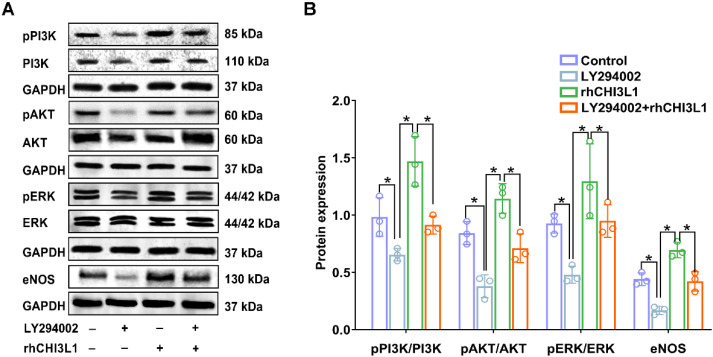
rhCHI3L1 promotes angiogenesis through the PI3K-AKT/ERK-eNOS signaling pathway in human umbilical vein endothelial cells (HUVEC). (**A**) Representative pictures of western blotting for detection of total and phosphorylated PI3K, AKT, ERK as well as eNOS protein expression in HUVEC. (**B**) Bar graph shows densitometric analysis of western blotting bands (*n* = 3/group). *Abbreviations:* rhCHI3L1, recombined human chitinase-3-like protein 1; LY294002, 2-morpholino-8-phenyl-4H-chromen-4-one. Symbol * indicates *p* < 0.05 between the relevant groups.

To investigate whether CHI3L1 promotes angiogenesis through the PI3K-AKT/ERK signaling pathway, HUVECs were co-incubated with a PI3K inhibitor (LY294002) and rhCHI3L1. The rhCHI3L1-induced significant increase in the ratios of pPI3K/PI3K, pAKT/AKT, pERK/ERK and eNOS/GAPDH were blocked by LY294002 (*p* < 0.05, Figure 7), indicating a cause-and-effect relationship between CHI3L1-induced activation of the PI3K-AKT/ERK signaling pathway and enhancement of angiogenesis.

**Figure 8:**
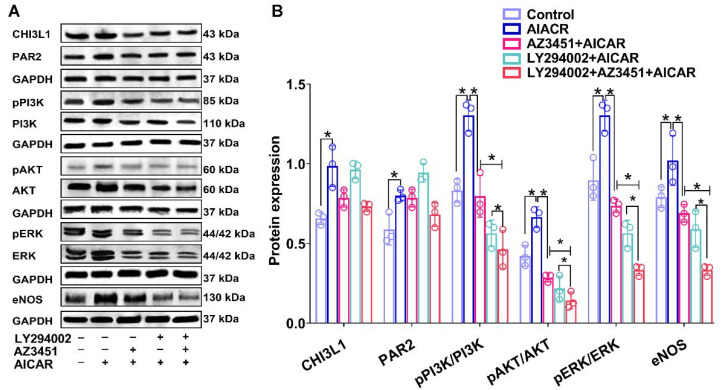
Exercise memetic—AICAR promotes angiogenesis through CHI3L1/PAR2-PI3K-AKT/ERK-eNOS signaling pathway in human umbilical vein endothelial cells (HUVEC). (**A**) Representative pictures of western blotting for detection of CHI3L1, PAR2, total and phosphorylated PI3K, AKT, ERK, and eNOS protein expression in HUVEC under various drug treatments. (**B**) Bar graph shows densitometric analysis of western blotting bands (*n* = 3/group). *Abbreviations:* LY294002—2-morpholino-8-phenyl-4H-chromen-4-one; AICAR—5-aminoimidazole-4-carboxamide-1-β-D-ribofuranoside; Symbol * indicates *p* < 0.05 between the relevant groups that are compared.

We further examined if the exercise mimetic AICAR would promote angiogenesis by activating CHI3L1/PAR2 in HUVECs. The ratios of pPI3K/PI3K, pAKT/AKT, pERK/ERK, and eNOS/GAPDH were significantly increased in AICAR group (*p* < 0.05 versus Control group, Figure 8) and co-incubation with AICAR and a PAR2 receptor blocker (AZ3451) reduced the AICAR-induced increase in the ratios of pPI3K/PI3K, pAKT/AKT, pERK/ERK, and eNOS/GAPDH (*p* < 0.05; Figure 8), suggesting in vitro stimulation of exercise promotes angiogenesis via activation of the CHI3L1/PAR2-PI3K-AKT/ERK signaling cascade.

## 4. Discussion

Adequate exercise training in patients recovering from MI can promote adaptive and beneficial changes in cardiac morphology and function, such as alleviated myocardial cell hypertrophy, apoptosis, and inflammation, as well as improved myocardial contractility and pumping function [27,28]. Despite the traditional caution on the risk of exercise-induced adverse events in patients with acute MI, recent studies have increasingly suggested that a lack of physical activity after MI would have little benefits in cardiac function, rather have negative effects on skeletal muscle, such as causing muscle atrophy [29]. The most salient result of our present study is the fact that four weeks of aerobic exercise after MI significantly increased protein expression and mRNA levels of CHI3L1 and its receptor PAR2 in the border zone of MI (Figure 4) and significantly improved post-MI ventricular function and reduced myocardial fibrosis (Figure 2). Post-MI exercise training also elevated serum levels of CHI3L1 (Figure 5), which are most likely originated from exercising skeletal muscles.

Additionally, our results confirmed that the previously reports showing that exercise training promoted capillary growth and angiogenesis in the heart [30,31,32]. Although the mechanism of exercise-induced angiogenesis is not fully understood, the beneficial effects seem to be closely related to changes in hemodynamics, such as increase in blood flow and/or venous return, as well as upregulation of genes and proteins that are responsible for angiogenesis in vascular endothelial cells. Elevated blood flow changes and shear stress on the vessel wall after exercise can activate PI3K-AKT-eNOS and improve endothelial function [33]. Exercise training can also promote cardiovascular angiogenesis [34,35,36]. Myocardial inflammation and oxidative stress are activated after MI, which result in cardiac cell necrosis and apoptosis and, in turn, lead to ventricular contractile dysfunction. Improving angiogenesis in patients with MI is one of the important ways to alleviate MI-induced cardiac injury and dysfunction and to promote post-MI rehabilitation of the injured heart [37]. The present study confirmed that post-MI exercise training significantly reduced cardiac cell apoptosis and increased angiogenesis in the rat hearts. We also demonstrated in in vitro models in HUVECs that the protein expression of CHI3L1 and its receptor PAR2 were significantly upregulated after 24 h of incubation with AICAR, an exercise memetic. HUVEC tubule formation assays also confirmed that rhCHI3L1 or AICAR significantly increased tubule formation. Furthermore, we also provided, for the first time, a complete array of evidence for the involvement of CHI3L1-PAR2-PI3K-AKT/ERK-eNOS signaling pathway in post-MI exercise-induced cardioprotection and the potential cause-and-effect relationship between CHI3L1-PAR2 signaling and exercise-induced cardioprotection was also demonstrated using PAR2 receptor blocker.

As abovementioned, CHI3L1 (YKL-40) has been studied as a circulating biomarker for several major diseases. A recent study measured plasma concentration of YKL-40 among 359 initially healthy women who subsequently developed cardiovascular events as compared with 359 age-matched female participants who remained free of disease during 17 years of follow-up. YKL-40 levels were higher in women with hypertension, diabetes, and obesity and correlated modestly with high-density lipoprotein cholesterol, triglycerides, and hsCRP, but not with low-density lipoprotein cholesterol. Baseline YKL-40 level was significantly associated with incident thromboembolic stroke, but no significant association was observed between YKL-40 and incident MI [38]. Other researchers found that in the general population, elevated plasma YKL-40 levels are associated with increased risk of ischemic stroke and ischemic cerebrovascular disease, independent of plasma CRP levels [39]. Serum YKL-40 is also increased in patients with slow coronary flow [40]. Interestingly, the results of our present study in male rats showed no significant increase in serum levels of CHI3L1 following MI, whereas post-MI exercise training led to enhanced serum levels of CHI3L1 (Figure 5). To our best knowledge, this is the first report on serum levels of CHI3L1 (YKL-40) in a rat model of MI with or without post-MI exercise. Our results suggested a beneficial association between the elevated serum CHI3L1 levels and cardioprotection afforded by post-MI exercise training.

Nevertheless, the current work has several limitations. First, we replied on a single PAR2 receptor blocker to demonstrate the role of CHI3L1-PAR2 signaling and as for any drug, the question on the specificity and potential off-target effects of the PAR2 receptor blocker can be raised. Future studies with more specific approaches to block CHI3L1-PAR2 signaling, such as PAR2 knockout or CHI3L1 knockout mice, may be helpful to validate the present results. In addition, our approaches to use cultured HUVEC are likely representing big vessel endothelial cells, which are different from myocardium angiogenesis that often occurs in micro-vasculatures. Future validation with microvascular endothelial cells may be needed to address any possibility that endothelial cells from the macro- and micro-vessels may respond to MI and/or exercise differently. In addition, direct visualization of cardiac vessels and measurement of blood flow to the heart would provide concrete evidence for improved angiogenesis following post-MI exercise training. Additional endothelial cell markers such as CD-31 and VE-cadherin may also help to validate the cell type specific observations.

After all, the potential translational significance of the present study appears on two fronts. First, this study has provided both in vivo and in vitro evidence to identify a key signaling role for myokine CHI3L1 and its receptor PAR2 in promoting exercise training-elicited revascularization in the post-MI myocardium, which represents a new mechanistic explanation for a previously revealed phenomenon on cardiac angiogenesis-promoting effects of exercise in post-MI failing rat hearts [41]. Second, these new mechanistic insights may have potential clinical implications, not only for rationalizing the well-controlled post-MI exercise training as a useful cardiac rehabilitation modality, but also for new drug development, pre-clinical studies, and clinical trials targeting the CHI3L1/PAR2 pathway. For example, to test if PAR2 agonists could serve as pharmaceutical memetics of exercise to enhance post-MI cardiac function and angiogenesis. Interestingly, mast cell tryptase, a known PAR2 agonist, selectively induced endothelial expression of the angiogenic chemokines CCL2/MCP-1 and CXCL8/IL-8 in the post-MI canine myocardium [42]. Therefore, selective stimulation of angiogenic chemokines via PAR2 agonist may be a candidate approach for healing post-MI heart.

## 5. Conclusions

The present study provided the first pieces of evidence for a post-MI exercise training-induced upregulation of both cardiac expression and circulating levels of CHI3L1 and its receptor PAR2, which in turn activate the PI3K-AKT/ERK signaling pathways, which lead to improvement of left ventricular function, and promotion of angiogenesis. These results may represent a novel mechanistic explanation for cardioprotective effects of post-MI exercise training and is warranted for further investigation and validation.

## Data Availability

All data needed to evaluate the conclusions in the paper have been provided in this article.
